# Step-Based Metrics and Overall Physical Activity in Children With Overweight or Obesity: Cross-Sectional Study

**DOI:** 10.2196/14841

**Published:** 2020-04-28

**Authors:** Jairo H Migueles, Cristina Cadenas-Sanchez, Elroy J Aguiar, Pablo Molina-Garcia, Patricio Solis-Urra, Jose Mora-Gonzalez, Eduardo García-Mármol, Eric J Shiroma, Idoia Labayen, Palma Chillón, Marie Löf, Catrine Tudor-Locke, Francisco B Ortega

**Affiliations:** 1 PROFITH (PROmoting FITness and Health through physical activity) Research Group Department of Physical and Sports Education, Faculty of Sport Sciences University of Granada Granada Spain; 2 Department of Kinesiology University of Massachusetts Amherst Amherst, MA United States; 3 Department of Rehabilitation Sciences Katholieke Universiteit Leuven Leuven Belgium; 4 IRyS (Investigación en Rendimiento Y Salud) Research Group School of Physical Education Pontificia Universidad Católica de Valparaíso Valparaíso Chile; 5 Laboratory of Epidemiology and Population Science National Institute on Aging National Institutes of Health Bethesda, MD United States; 6 Institute for Innovation and Sustainable Development in the Food Chain (IS-FOOD) Public University of Navarra Pamplona Spain; 7 Department of Biosciences and Nutrition Karolinska Institutet Huddinge Sweden; 8 Department of Medical and Health Sciences Linköping University Linköping Sweden

**Keywords:** motion sensor, pedometer, sedentary behavior, MVPA, cadence

## Abstract

**Background:**

Best-practice early interventions to increase physical activity (PA) in children with overweight and obesity should be both feasible and evidence based. Walking is a basic human movement pattern that is practical, cost-effective, and does not require complex movement skills. However, there is still a need to investigate how much walking—as a proportion of total PA level—is performed by children who are overweight and obese in order to determine its utility as a public health strategy.

**Objective:**

This study aimed to (1) investigate the proportion of overall PA indicators that are explained by step-based metrics and (2) study step accumulation patterns relative to achievement of public health recommendations in children who are overweight and obese.

**Methods:**

A total of 105 overweight and obese children (mean 10.1 years of age [SD 1.1]; 43 girls) wore hip-worn accelerometers for 7 days. PA volumes were derived using the daily average of counts per 15 seconds, categorized using standard cut points for light-moderate-vigorous PA (LMVPA) and moderate-to-vigorous PA (MVPA). Derived step-based metrics included volume (steps/day), time in cadence bands, and peak 1-minute, 30-minute, and 60-minute cadences.

**Results:**

Steps per day explained 66%, 40%, and 74% of variance for counts per 15 seconds, LMVPA, and MVPA, respectively. The variance explained was increased up to 80%, 92%, and 77% by including specific cadence bands and peak cadences. Children meeting the World Health Organization recommendation of 60 minutes per day of MVPA spent less time at zero cadence and more time in cadence bands representing sporadic movement to brisk walking (ie, 20-119 steps/min) than their less-active peers.

**Conclusions:**

Step-based metrics, including steps per day and various cadence-based metrics, seem to capture a large proportion of PA for children who are overweight and obese. Given the availability of pedometers, step-based metrics could be useful in discriminating between those children who do or do not achieve MVPA recommendations.

**Trial Registration:**

ClinicalTrials.gov NCT02295072; https://clinicaltrials.gov/ct2/show/NCT02295072

## Introduction

Decreased physical activity (PA) is associated with increased risk of noncommunicable diseases [1-3] and is responsible for approximately 9% of premature mortality [4]. Worldwide PA deficits [2,5-7] and inequalities between countries regarding PA levels [8] require effective counteractive strategies, especially in populations at risk such as people with overweight and obesity. For example, evidence [9,10] suggests that low levels of PA initiated in childhood and perpetuated in adulthood set up adults with overweight and obesity for an increased array of comorbidities during their life span [11]. Best-practice early interventions should be both feasible and evidence based. Walking is a basic human movement pattern that is practical, cost-effective, and does not require complex movement skills. Thus, focusing on ambulatory activity could be the most accessible strategy to increase PA levels in children with overweight and obesity [12] who do not engage frequently in sports [13] and present poorer movement skills than normal-weight children [14]. However, there is still a need to investigate how much ambulatory activity is performed by children with overweight and obesity, as proportion of total PA level, in order to determine its utility as a public health strategy. Information on what type of PA children with overweight and obesity are more likely to perform could help to plan more effective public health strategies, since intervening on a behavior that is frequently occurring (eg, walking) would have a greater impact than generating a new behavior.

The ability to study health-related influences of PA has advanced in parallel with the increased use of accelerometer-based wearable technologies [15]. Accelerometers are capable of detecting human movement, but are primarily sensitive to ambulatory activity, the most common form of PA performed by adults [16,17]. However, children’s movement patterns may be more variable and less is known about how predominant ambulatory activity, primarily walking, is relative to other types of PA behaviors. Time-stamped accelerometers are capable of detecting step-based metrics, including a tally of step accumulation over the day (ie, volume [steps/day]), the time spent in incremental cadence bands (eg, time spent walking at 80-99 steps/min), and/or peak 1-minute, 30-minute, and 60-minute cadence indices (ie, average steps/min of the highest 1, 30, or 60 nonconsecutive minutes in a day, respectively) [18-20]. Collectively, these metrics are referred to hereafter as step-based metrics.

Therefore, this study aimed to (1) investigate the proportion of overall PA that is explained by ambulatory activity (ie, step-based metrics) in children with overweight and obesity and (2) study step-based patterns relative to PA guidelines achievement in children with overweight and obesity.

## Methods

### Study Design and Participants

This cross-sectional analysis included data collected during the baseline assessment of the ActiveBrains project [21]. A detailed description of the study design, inclusion criteria, and methods have been published elsewhere [22]. Briefly, ActiveBrains is a randomized controlled trial intended to examine the effect of a 20-week PA intervention on brain structure, brain function, cognitive performance, academic achievement, and physical and mental health outcomes in children with overweight and obesity [22]. A total of 110 children (8.5-11 years old) with overweight and obesity, defined according to the World Obesity Federation cut points [23,24], were recruited from Granada, Spain. Data were collected from November 2014 to February 2016. Parents or legal guardians were informed of the purpose of the study and written informed parental consent was obtained. The ActiveBrains project was approved by the Human Research Ethics Committee of the University of Granada and was registered as a clinical trial at ClinicalTrials.gov (NCT02295072).

### Procedures

As part of the protocol of the ActiveBrains project [22], body weight and height were measured to the nearest 0.1 kg and 0.1 cm using a seca 861 electronic scale (seca gmbh & co kg) and a seca 225 precision stadiometer (seca gmbh & co kg), respectively. BMI (kg/m^2^) was then calculated. Overweight and obesity were classified based on the cutoffs of the World Obesity Federation [23].

Participants’ overall PA and step-based metrics were measured with a GT3X+ accelerometer (ActiGraph) worn on their right hip for 7 complete days (24-hour wear-time protocol). Participants were encouraged to wear the accelerometers as many hours as possible and only remove them for water activities (ie, shower or swimming). Concurrently, participants logged the time they went to bed and woke up in a diary for the entire 7 days. All participants with at least 4 days, including 1 weekend day, with 16 hours or more of accelerometer wear time were included in the analyses (N=105).

### Data Reduction

Raw .gt3x files (100 Hz) were loaded and processed with the ActiLife software (ActiGraph) to obtain activity counts (ie, metric intended to capture body movement), accumulated in the vertical axis over 15-second epochs, and steps accumulation over 60-second epochs using the default filter developed by ActiGraph. Nonwear time was detected based on the raw acceleration values of the three axes using a previously published algorithm [25]. Briefly, each 15-minute block was classified as nonwear time if the standard deviation of two out of the three axes was lower than 13 mg during the surrounding 60-minute moving window, or if the mean acceleration for two out of the three axes was lower than 50 mg. Likewise, sustained abnormally high accelerations (ie, higher than 5.5 g; assumed to be related to device malfunction) were detected and labelled as nonwear time. The identified nonwear time, including sustained abnormally high accelerations, was imputed with the mean acceleration value for the corresponding time period over the remaining days of recording. Sleeping hours were identified using an automated algorithm guided by participants’ logged times [26] and excluded from analyses. Nonwear time and sleeping hours identification were performed using functions included in the R package GGIR (The R Foundation) [25,27].

Each 15-second epoch was classified into sedentary time or time at different PA intensities using the activity-count cut points developed by Evenson et al [28]. Specifically, these were as follows: sedentary time (≤25 counts/15 sec), light intensity (26-573 counts/15 sec), moderate intensity (574-1002 counts/15 sec), and vigorous intensity (≥1003 counts/15 sec). Daily average acceleration (counts/15 sec), time spent at light-moderate-vigorous PA (LMVPA; >25 counts/15 sec), and time spent at moderate-to-vigorous PA (MVPA) intensity (>573 counts/15 sec) were included in the analyses as indicators of overall PA. Daily average acceleration (counts/15 sec) and MVPA are indicators commonly used to represent overall PA [29-31]. LMVPA was also included following the recommendations of the 2018 Physical Activity Guidelines Advisory Committee Scientific Report, which acknowledge the importance of any kind of PA for health [32]. Furthermore, light PA could be a stimulus worthy to consider in children with overweight and obesity since they usually engage in insufficient MVPA.

Total ambulatory activity volume was derived as the number of steps per day. Furthermore, ambulatory activity cadence patterns were estimated as described previously for adults [33] and children [19]. Briefly, cadences were organized into bands of approximately 20 steps per minute increments. These cadence bands have been previously associated with the following behavioral descriptors: incidental movement (1-19 steps/min), sporadic movement (20-39 steps/min), purposeful movement (40-59 steps/min), slow walking (60-79 steps/min), medium walking (80-99 steps/min), brisk walking (100-119 steps/min), and faster walking (≥120 steps/min). Time spent in each one of these bands, as well as time at zero cadence (TZC), were computed. In addition, the peak 60-minute, peak 30-minute, and peak 1-minute cadences were computed by rank-ordering each participants’ data for each day and then computing the average steps per minute for the top 60, 30, and 1 minute, respectively. The ActiGraph GT3X+ accelerometer has been demonstrated to be valid for counting steps [34,35] and its identified cadence bands have been used to describe cadence patterns in large cohorts [19]. Mean daily counts per 15 seconds, sedentary time, and time-based and step-based metrics were then calculated as follows:

(mean of available weekdays × 5) + (mean of available weekend days × 2) / 7

### Data Analyses

Descriptive characteristics of participants were presented as means and SD. We used simple linear regression models to study the proportion of overall PA indicators explained by each step-based metric, and stepwise regression models to study the proportion explained by using several step-based metrics as predictors. First, the variable that explained the highest proportion of the outcome variance was introduced. Then, those variables that significantly increased the proportion of variance explained were introduced. If any of the variables presented a variance inflation factor above 7, it was excluded from the model. TZC was not included in these models since it represents inactivity. In addition, we identified those participants who achieved the World Health Organization PA recommendations for this age group [36] (ie, at least 60 min/day of MVPA). Two-sample *t* tests were then used to compare time spent in different cadence bands; the peak 60-minute, the peak 30-minute, and the peak 1-minute cadences of children who accomplished the PA recommendations were also compared, using two-sample *t* tests, with their peers who did not. All analyses were performed in R [37]. The significance level was set at *P*<.05.

## Results 

### Descriptive Characteristics

Table 1 presents anthropometric characteristics; sedentary time; light, moderate, and vigorous PA; as well as step-based metrics for all participants stratified by sex.

Multimedia Appendix 1 (Table A1) shows the same descriptive characteristics stratified by weight status group (ie, overweight, mild obesity, severe obesity, and morbid obesity).

**Table 1:** Anthropometry, sedentary time, time-based physical activity (PA) metrics, and step-based metrics of overweight and obese children.

Characteristic		All participants (N=105), mean (SD)	Boys (n=62), mean (SD)	Girls (n=43), mean (SD)
Age (years)		10.1 (1.1)	10.2 (1.2)	9.9 (1.1)
**Anthropometry**				
	Weight (kg)	56.6 (11.1)	57.4 (11.1)	55.4 (11.1)
	Height (cm)	144.4 (8.3)	145.0 (7.8)	143.6 (8.9)
	BMI (z-score)	3.03 (0.87)	3.19 (0.97)	2.81 (0.64)
**Awake and wear time (min/day)**				
	Awake time	919.6 (31.5)	921.3 (28.7)	917.0 (35.2)
	Wear time during waking	903.1 (35.3)	905.1 (32.7)	900.4 (39.0)
**Sedentary time and PA intensities (min/day)**				
	Sedentary time	600.8 (69.6)	593.6 (69.1)	611.1 (69.9)
	Light-intensity PA	273.2 (51.7)	276.4 (51.2)	268.5 (52.5)
	Moderate-intensity PA	34.0 (11.6)	37.9 (12.4)	28.2 (7.4)
	Vigorous-intensity PA	10.7 (6.7)	12.3 (7.5)	8.3 (4.4)
	Moderate-to-vigorous PA	44.7 (16.8)	50.3 (18.2)	36.6 (10.3)
**Step-based metrics**				
	Volume (steps/day)	8676.8 (2202.9)	9257.6 (2431.9)	7836.9 (1485.4)
	Peak 60-minute cadence (steps/min)	63.7 (13.6)	66.3 (14.4)	59.8 (11.4)
	Peak 30-minute cadence (steps/min)	78.0 (14.5)	79.7 (15.2)	75.4 (13.2)
	Peak 1-minute cadence (steps/min)	111.5 (13.3)	111.2 (13.3)	111.8 (13.3)
**Time spent at different cadence bands (min/day)**				
	0 steps/minute	346.6 (78.1)	343.0 (79.5)	351.7 (76.7)
	1-19 steps/minute	439.0 (63.4)	434.9 (62.2)	444.8 (65.4)
	20-39 steps/minute	71.9 (18.2)	73.5 (19.1)	69.7 (16.8)
	40-59 steps/minute	27.5 (9.2)	30.3 (9.8)	23.6 (6.6)
	60-79 steps/minute	15.9 (7.9)	18.6 (8.5)	12.0 (4.6)
	80-99 steps/minute	10.2 (6.2)	11.8 (7.0)	8.0 (3.8)
	100-119 steps/minute	6.6 (6.0)	7.4 (7.0)	5.5 (4.0)
	≥120 steps/minute	1.6 (2.6)	1.8 (2.7)	1.3 (2.3)

### Proportion of Total Physical Activity Explained by Step-Based Metrics

**Figure 1:**
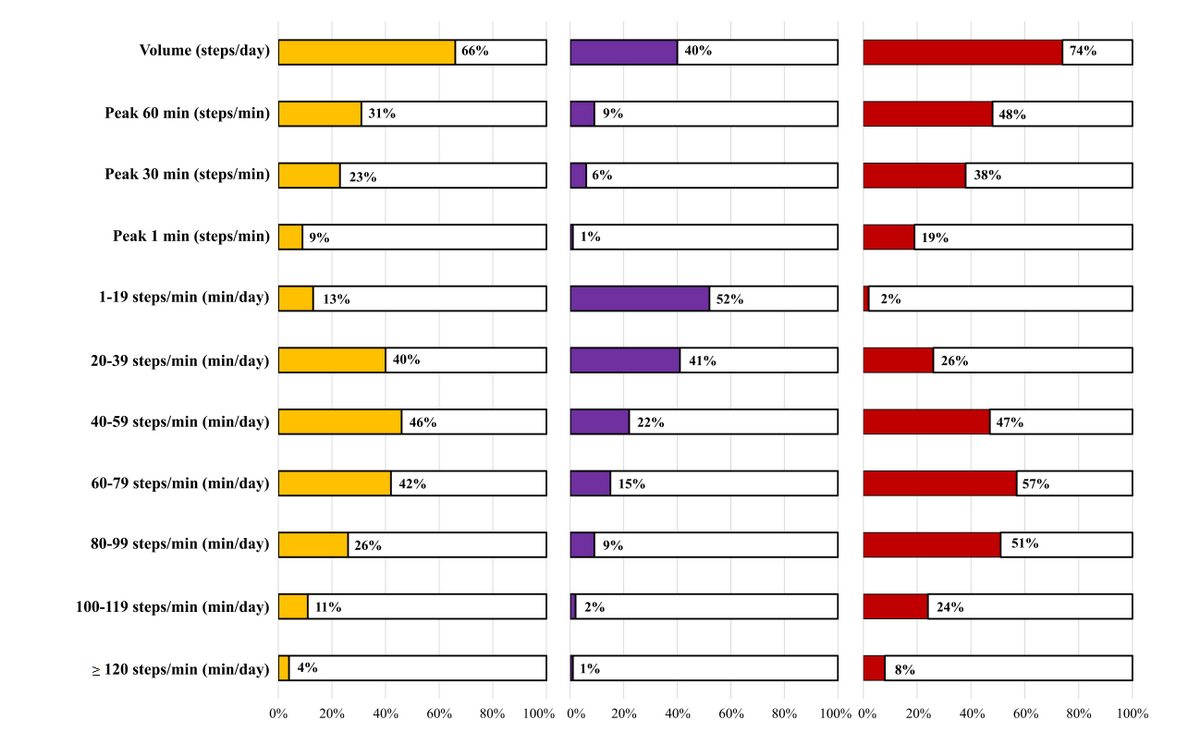
Proportion of variance (*r*^2^) in overall physical activity indicators, which is explained independently by each step-based metric. LMVPA: light-moderate-vigorous physical activity; MVPA: moderate-to-vigorous physical activity.

Figure 1 depicts the proportion of variance in indicators of overall PA (ie, counts/15 sec, LMVPA, and MVPA) that each step-based metric explained (*r*^2^) in separate linear regression models (ie, simple linear regression with each step-based metric as predictor and overall PA metric as outcome). Among the step-based metrics, steps per day explained the highest proportion of counts per 15 seconds and MVPA (66% and 74%, respectively), while time at 1-19 steps per minute explained the highest proportion of LMVPA (52%). Overall, peak cadence indicators explained a lower proportion of the variance than steps per day in overall PA indicators. Likewise, the shorter the time intervals used to calculate the specific peak cadence indicator, the lower the explanation capacity of the metric, which is to be expected given the shorter time frame represented (ie, 60 min > 30 min > 1 min).

**Figure 2:**
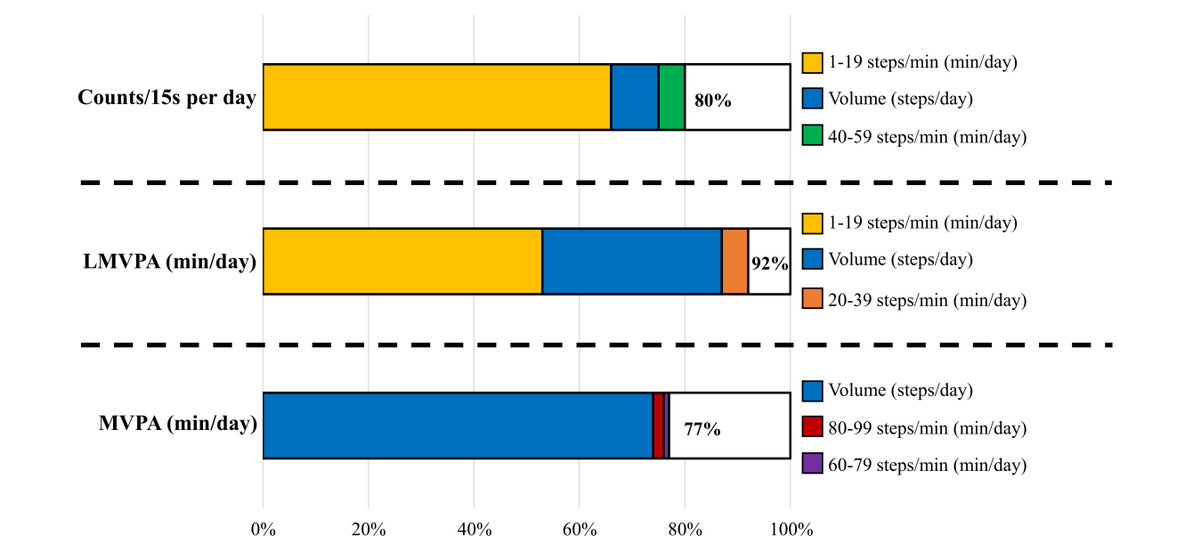
Proportion of variance (*r*^2^) in overall physical activity (PA) indicators (ie, counts/15 sec, light-moderate-vigorous PA [LMVPA], and moderate-to-vigorous PA [MVPA]), which is explained by a combination of step-based metrics, calculated using stepwise linear regressions. All predictors presented variance inflation factors of <6 in the selected models.

### Cadence Patterns According to Physical Activity Guidelines

**Figure 3:**
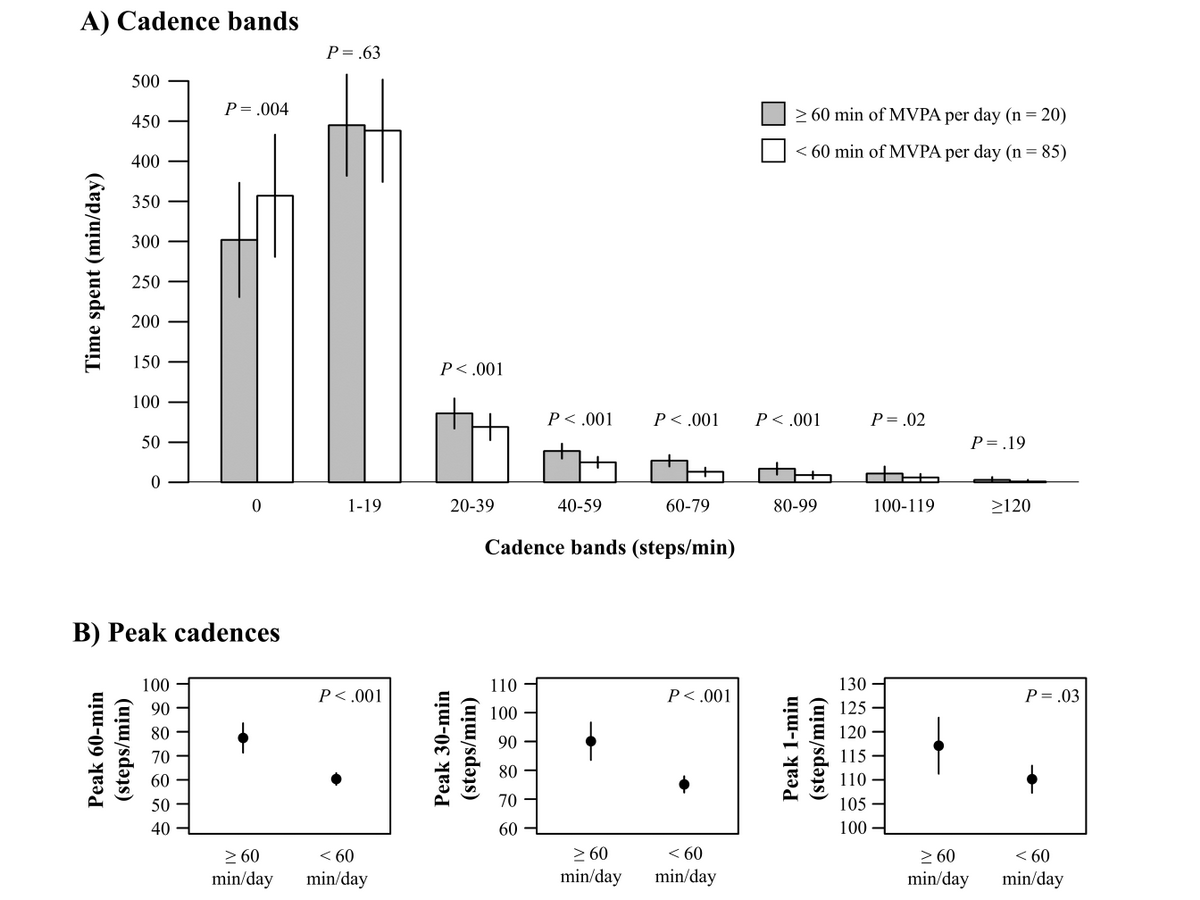
Time spent in each cadence band (panel A) and peak cadences (panel B) across children meeting or not meeting the physical activity guidelines (ie, ≥60 minutes of MVPA/day). Error bars represent SDs. MVPA: moderate-to-vigorous physical activity.

Out of the 105 children, 20 (19.0%) achieved the recommended amount of MVPA (ie, ≥60 min/day). Children who performed less than 60 minutes per day of MVPA also had significantly higher values for TZC (*P*=.004) and less time in cadence bands from 20 to 120 steps per minute, compared with those who performed 60 minutes per day or more of MVPA (all *P*<.02) (see Figure 3). Likewise, peak 60-minute, peak 30-minute, and peak 1-minute cadences were higher in children who achieved the 60 minutes per day of MVPA.

**Figure 4:**
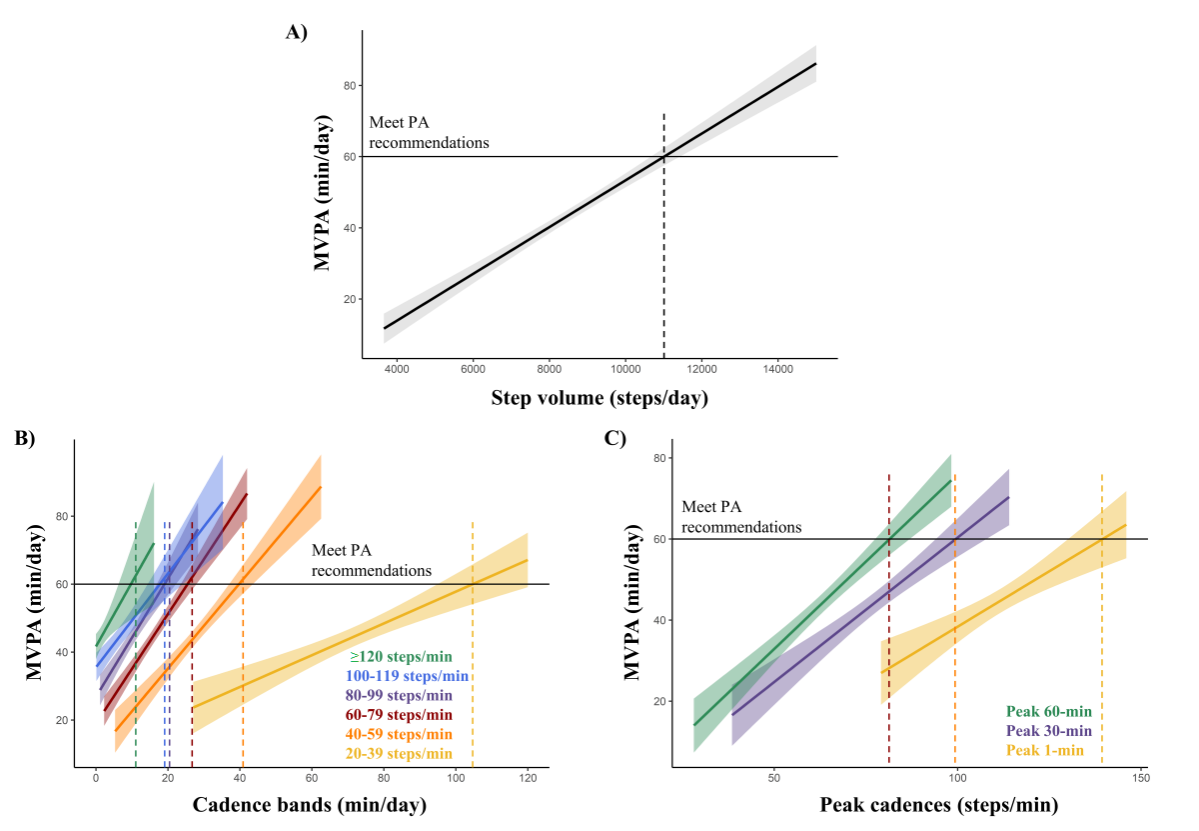
Linear regression line, with 95% CI (shaded areas), for the association between moderate-to-vigorous physical activity (MVPA) and step-based metrics: step volume (panel A), cadence bands (panel B), and peak cadences (panel C). PA: physical activity.

Specifically, participants had to walk around 11,000 steps per day to achieve the recommended dose of MVPA (see Figure 4, panel A). Likewise, they had to spend 105 minutes per day walking at 20-39 steps per minute, 40 minutes per day at 40-59 steps per minute, 25 minutes per day at 60-79 steps per minute, 19 minutes per day at 80-99 steps per minute, 18 minutes per day at 100-119 steps per minute, or 10 minutes per day at or above 120 steps per minute (see Figure 4, panel B). Finally, their peak cadences had to be higher than 140 steps per minute for peak 1-minute cadence, 100 steps per minute for peak 30-minute cadence, or 80 steps per minute for peak 60-minute cadence (see Figure 4, panel C).

## Discussion

The main findings of this study are as follows:

1. Steps per day and the 1-19 steps per minute cadence band explained the greatest amount of overall PA (ie, counts/15 sec, 66%; LMVPA, 52%; and MVPA, 74%) in children with overweight and obesity. The proportion of variance explained was further improved—by up to 77%-92%—by adding other step-based metrics to the models.
2. Cadence-based step patterns significantly differed between those children with overweight and obesity who achieved the PA recommendations compared with those who did not. Together, these findings seem to point out ambulatory activity as a major source of PA in children with overweight and obesity, as it has been previously reported in adults [16,17].

Further studies with larger and more representative samples should corroborate this finding. This finding can be leveraged to design appropriate PA interventions (ie, by investigating the amount of walking at a certain intensity needed to meet PA recommendations) as a strategy to lower lifespan health risks in this vulnerable population.

Steps per day and the 1-19 steps per minute cadence band were the best explanatory factors of overall PA. Specifically, more than half of the variation in overall PA could be explained by either steps per day or the 1-19 steps per minute cadence band in children with overweight and obesity, depending on the overall PA indicator regressed (ie, 66% for counts/15 sec [steps/day], 52% for LMVPA [1-19 steps/min], and 74% for MVPA [steps/day]). Furthermore, all of the stepwise models included steps per day to estimate either counts per 15 seconds, LMVPA, or MVPA. Accordingly, our sample was active for 5.3 hours per day (ie, LMVPA), during which time they spent around 2.2 hours per day in ambulatory activity (ie, from sporadic movement to faster cadences). This presumes around 40% of the time spent was in LMVPA, which is similar to the estimation obtained to predict LMVPA from steps per day (ie, 40%).

However, steps per day was not the only important factor in the prediction of overall PA indicators. Information regarding step accumulation pattern increased the prediction capacity up to 80%, 92%, and 77%, for counts per 15 seconds, LMVPA, and MVPA, respectively. These findings support the concept of also considering stepping rate, which has been associated with health-related intensity levels in children [20] in addition to steps per day. The fact that the explanation of the variability of LMVPA increased substantially (ie, from 40% to 92%) by including more metrics in the stepwise models is noteworthy. This suggests that considering both steps per day and certain cadence bands we can explain around 90% of their active minutes. However, the explanation of the variability of MVPA only increased from 74% to 77%, which means that almost all information on MVPA is already provided by steps per day. Together, it seems clear that step-based metrics are more powerful in explaining light-intensity PA than higher intensities.

To this end, Tudor-Locke et al found that walking at 115 steps per minute requires an energy expenditure of approximately 4 metabolic equivalents (ie, moderate PA intensity for children) in 9-11-year-old children, measured while walking on a treadmill [38]. However, the cadence-intensity relationship observed under laboratory-controlled conditions may not be generalizable to free-living data from children with overweight and obesity. Likewise, caution is advised since measurement tools differed between studies (ie, direct observation vs accelerometers). We observed around 34 minutes per day classified as moderate PA intensity by Evenson et al cut points [28] and, in turn, around only 2 minutes per day (SD 3) accumulated at a cadence of more than 115 steps per minute, which is indicative of MVPA intensity in this age group as measured in lab conditions [38]. A source for this difference could be the epoch length used to derive moderate intensity [39] (ie, 15 sec for Evenson et al cut points and 60 sec for time spent above 100 steps/min). Estimations based on Evenson cut points could be able to capture short bouts of moderate PA up to 15 seconds, while step-based estimations of moderate PA are limited to those bouts lasting at least 1 minute. We decided to use 60-second epochs for cadence to maintain consistency with previous studies, to ease comparability of findings and because there are no studies examining the cadence measured in 15-second epochs and intensity to date, making it more difficult to interpret the findings. It could be also argued that most of the moderate PA performed by our sample was not related to ambulatory activity, which seems unlikely because step-based metrics explained almost 80% of the variance in MVPA. We must also acknowledge that metabolic intensity is indirectly inferred from detected movement signals and is not a clear indicator of metabolic cost, so there are likely to be measurement differences attributable to differential definitions. Therefore, further research is needed to understand how free-living cadence bands relate to energy expenditure and accelerometer signals.

Peak cadence indices and cadence bands have been previously used as proxy indicators for ambulatory activity intensity and pattern, respectively, in children [19] and adults [33,40]. In congruence with Barreira et al [19], we found that most of the day was spent in low intensity or sedentary behaviors. Specifically, we found around 10 hours per day of sedentary time using Evenson et al [28] cut points or, in regard to step-based metrics, a TZC value of 5.8 hours per day and 7.3 hours per day in incidental movements (1-19 steps/min). Barreira et al reported similar step accumulation patterns in 6-11-year-old children from the US National Health and Nutrition Examination Survey (NHANES 2005-2006). Notably, only 38% of the NHANES population−based sample were overweight and obese [19]. In contrast, our sample of children who were overweight and obese accumulated more TZC, as well as time in incidental movements (1-19 steps/min), and less time in cadence bands, from sporadic movement to faster walking (20-120 steps/min). Likewise, differences in accelerometer models, study design, and socioenvironmental context should be considered when comparing these studies.

According to the Evenson et al cut-points definition of MVPA [28], 20 children out of 105 (19.0%) from our sample met the PA recommendation of at least 60 minutes per day of MVPA [36]. This finding should be interpreted with caution since quantification of time-based PA with accelerometers is notoriously challenging and is dependent on a variety of data collection and processing decisions [39], including those related to selecting appropriate analytical cut points [41]. We have previously reported that changing cut points can derive extremely different estimations of the proportion of children meeting PA recommendations in this sample [41]. It is also important to consider that PA recommendations are mainly based on self-reported data, which could bias interpretation over objective data. Additionally, when compared to normative values from NHANES 2005-2006 [42], our sample can be considered *below average* for steps per day for 8-9-year-old children (ie, 7647-9398 steps/day) or *average* for 10-11-year-olds (ie, 8504-10,066 steps/day). Likewise, our sample presented *below average* values for peak 60-minute cadence (ie, 62-71 steps/min). Nevertheless, a large proportion of the count-based MVPA performed was related to step-based metrics, which suggests that limited ambulatory behaviors could be responsible for the low prevalence of children meeting the PA recommendations. Furthermore, we found significant differences in ambulatory activity intensity (ie, time spent in almost every band cadence was significantly different) between those who met and those who did not meet the PA recommendations. Specifically, children who met the recommendations spent around 55 minutes per day less in TZC, 17 minutes per day more in sporadic movement (ie, 20-39 steps/min), 14 minutes per day more in purposeful movement (ie, 40-59 steps/min), 13 minutes per day more in slow walking (ie, 60-79 steps/min), 8 minutes per day more in medium walking (ie, 80-99 steps/min), and 5 minutes per day more in brisk walking (ie, 100-119 steps/min). Additionally, peak 1-minute, 30-minute, and 60-minute cadences seem to be able to discern between children achieving or not achieving the recommended dose of MVPA per day (ie, 60 min/day).

Findings of this study have several practical implications to consider. Two examples are as follows:

1. As a large proportion of overall PA identified by accelerometers is explained by step-based metrics in children with overweight and obesity, these measures could be used to describe and compare PA patterns in this population.
2. It could be assumed that increasing ambulatory activity volume and intensity is a feasible form of PA that can increase the chances of meeting PA recommendations in this population. This is especially important to consider as ambulatory activity is a feasible PA strategy that may lead to several health benefits (eg, improved body composition and mental health) in children with overweight and obesity [43-45]. Notably, walking does not require complex movement skills, so it can be performed by most populations, including children with overweight and obesity who frequently do not engage in sports because of their low physical competence [14].

Several limitations should be acknowledged. First and foremost, accelerometer measurements of PA are influenced by a variety of data collection and processing decisions [39]. This means that it cannot be considered a gold standard for overall PA measurement and that changes in the quantification of PA could change the findings observed in this study. However, we were as consistent as possible regarding the measurement of both overall PA and step-based metrics. Both outcomes come from the same hip-worn accelerometer, and cut points used are based on the vertical axis acceleration, which is consistent with the ActiGraph procedures to detect steps. This would reduce the methodological inconsistencies between the overall PA and the step-based metrics estimations, which, in turn, can be considered as a strength of this study. Note that epoch length discrepancies between count-based and step-based metrics may be partially responsible for the differences observed. However, our findings should be interpreted with caution since overall PA refers to accelerometer-determined PA, which is not a gold standard and could ignore certain activities such as swimming. Note that step-based metrics derived from pedometers could vary the findings from this study and their relationship with overall PA should be investigated. Likewise, another strength to highlight is that we are focusing on a population who may benefit greatly from increases in ambulatory activity; for example, this study demonstrates that they could have substantially increased chances of meeting PA recommendations by only focusing on ambulatory activity.

In conclusion, step-based metrics including steps per day and various cadence-based intensity indicators seem to capture the majority of PA in children with overweight and obesity. Given that pedometers are more affordable than accelerometers, step-based metrics could be useful for discriminating between those children who do or do not achieve MVPA recommendations.

## Appendix

Multimedia Appendix 1 Anthropometry, sedentary time, time-based physical activity metrics, and step-based metrics of overweight and obese children stratified by weight status, according to the World Obesity Federation standards.

## Abbreviations

ERDF: European Regional Development Fund
FEDER: Fondo Europeo de DEsarrollo Regional
I+D+I: Investigación + Desarrollo + Innovación
ISCIII: Instituto de Salud Carlos III
LMVPA: light-moderate-vigorous physical activity
MINECO: Ministerio de Economía y COmpetitividad
MVPA: moderate-to-vigorous physical activity
NHANES: National Health and Nutrition Examination Survey
PA: physical activity
PN: Plan Nacional
RETICS: REdes Temáticas de Investigación Cooperativa en Salud
SAMID III: red de SAlud Materno Infantil y Desarrollo
TZC: time at zero cadence
UCEES: Unit of Excellence on Exercise and Health

## References

[1] Booth FW, Laye MJ, Lees SJ, Rector RS, Thyfault JP (2008). Reduced physical activity and risk of chronic disease: The biology behind the consequences. Eur J Appl Physiol.

[2] Forouzanfar MH, Alexander L, Anderson HR, Bachman VF, Biryukov S, Brauer M, Burnett R, Casey D, Coates MM, Cohen A, Delwiche K, Estep K, Frostad JJ, Astha KC, Kyu HH, Moradi-Lakeh M, Ng M, Slepak EL, Thomas BA, Wagner J, Aasvang GM, Abbafati C, Abbasoglu OA, Abd-Allah F, Abera SF, Aboyans V, Abraham B, Abraham JP, Abubakar I, Abu-Rmeileh NM, Aburto TC, Achoki T, Adelekan A, Adofo K, Adou AK, Adsuar JC, Afshin A, Agardh EE, Al KM, Al LF, Alam SS, Alasfoor D, Albittar MI, Alegretti MA, Aleman AV, Alemu ZA, Alfonso-Cristancho R, Alhabib S, Ali R, Ali MK, Alla F, Allebeck P, Allen PJ, Alsharif U, Alvarez E, Alvis-Guzman N, Amankwaa AA, Amare AT, Ameh EA, Ameli O, Amini H, Ammar W, Anderson BO, Antonio CA, Anwari P, Argeseanu CS, Arnlöv J, Arsenijevic VS, Artaman A, Asghar RJ, Assadi R, Atkins LS, Atkinson C, Avila MA, Awuah B, Badawi A, Bahit MC, Bakfalouni T, Balakrishnan K, Balalla S, Balu RK, Banerjee A, Barber RM, Barker-Collo SL, Barquera S, Barregard L, Barrero LH, Barrientos-Gutierrez T, Basto-Abreu AC, Basu A, Basu S, Basulaiman MO, Batis RC, Beardsley J, Bedi N, Bekele T, Bell ML, Benjet C, Bennett DA, Benzian H, Bernabé E, Beyene TJ, Bhala N, Bhalla A, Bhutta ZA, Bikbov B, Bin AA, Blore JD, Blyth FM, Bohensky MA, Bora BB, Borges G, Bornstein NM, Bose D, Boufous S, Bourne RR, Brainin M, Brazinova A, Breitborde NJ, Brenner H, Briggs AD, Broday DM, Brooks PM, Bruce NG, Brugha TS, Brunekreef B, Buchbinder R, Bui LN, Bukhman G, Bulloch AG, Burch M, Burney PG, Campos-Nonato IR, Campuzano JC, Cantoral AJ, Caravanos J, Cárdenas R, Cardis E, Carpenter DO, Caso V, Castañeda-Orjuela CA, Castro RE, Catalá-López F, Cavalleri F, Çavlin A, Chadha VK, Chang J, Charlson FJ, Chen H, Chen W, Chen Z, Chiang PP, Chimed-Ochir O, Chowdhury R, Christophi CA, Chuang T, Chugh SS, Cirillo M, Claßen TK, Colistro V, Colomar M, Colquhoun SM, Contreras AG, Cooper C, Cooperrider K, Cooper LT, Coresh J, Courville KJ, Criqui MH, Cuevas-Nasu L, Damsere-Derry J, Danawi H, Dandona L, Dandona R, Dargan PI, Davis A, Davitoiu DV, Dayama A, de Castro EF, De la Cruz-Góngora V, De Leo D, de Lima G, Degenhardt L, del Pozo-Cruz B, Dellavalle RP, Deribe K, Derrett S, Des Jarlais DC, Dessalegn M, deVeber GA, Devries KM, Dharmaratne SD, Dherani MK, Dicker D, Ding EL, Dokova K, Dorsey ER, Driscoll TR, Duan L, Durrani AM, Ebel BE, Ellenbogen RG, Elshrek YM, Endres M, Ermakov SP, Erskine HE, Eshrati B, Esteghamati A, Fahimi S, Faraon EJ, Farzadfar F, Fay DF, Feigin VL, Feigl AB, Fereshtehnejad S, Ferrari AJ, Ferri CP, Flaxman AD, Fleming TD, Foigt N, Foreman KJ, Paleo UF, Franklin RC, Gabbe B, Gaffikin L, Gakidou E, Gamkrelidze A, Gankpé FG, Gansevoort RT, García-Guerra FA, Gasana E, Geleijnse JM, Gessner BD, Gething P, Gibney KB, Gillum RF, Ginawi IA, Giroud M, Giussani G, Goenka S, Goginashvili K, Gomez DH, Gona P, Gonzalez DC, González-Castell D, Gotay CC, Goto A, Gouda HN, Guerrant RL, Gugnani HC, Guillemin F, Gunnell D, Gupta R, Gupta R, Gutiérrez RA, Hafezi-Nejad N, Hagan H, Hagstromer M, Halasa YA, Hamadeh RR, Hammami M, Hankey GJ, Hao Y, Harb HL, Haregu TN, Haro JM, Havmoeller R, Hay SI, Hedayati MT, Heredia-Pi IB, Hernandez L, Heuton KR, Heydarpour P, Hijar M, Hoek HW, Hoffman HJ, Hornberger JC, Hosgood HD, Hoy DG, Hsairi M, Hu G, Hu H, Huang C, Huang JJ, Hubbell BJ, Huiart L, Husseini A, Iannarone ML, Iburg KM, Idrisov BT, Ikeda N, Innos K, Inoue M, Islami F, Ismayilova S, Jacobsen KH, Jansen HA, Jarvis DL, Jassal SK, Jauregui A, Jayaraman S, Jeemon P, Jensen PN, Jha V, Jiang F, Jiang G, Jiang Y, Jonas JB, Juel K, Kan H, Kany RS, Karam NE, Karch A, Karema CK, Karthikeyan G, Kaul A, Kawakami N, Kazi DS, Kemp AH, Kengne AP, Keren A, Khader YS, Khalifa SE, Khan EA, Khang Y, Khatibzadeh S, Khonelidze I, Kieling C, Kim D, Kim S, Kim Y, Kimokoti RW, Kinfu Y, Kinge JM, Kissela BM, Kivipelto M, Knibbs LD, Knudsen AK, Kokubo Y, Kose MR, Kosen S, Kraemer A, Kravchenko M, Krishnaswami S, Kromhout H, Ku T, Kuate DB, Kucuk BB, Kuipers EJ, Kulkarni C, Kulkarni VS, Kumar GA, Kwan GF, Lai T, Lakshmana BA, Lalloo R, Lallukka T, Lam H, Lan Q, Lansingh VC, Larson HJ, Larsson A, Laryea DO, Lavados PM, Lawrynowicz AE, Leasher JL, Lee J, Leigh J, Leung R, Levi M, Li Y, Li Y, Liang J, Liang X, Lim SS, Lindsay MP, Lipshultz SE, Liu S, Liu Y, Lloyd BK, Logroscino G, London SJ, Lopez N, Lortet-Tieulent J, Lotufo PA, Lozano R, Lunevicius R, Ma J, Ma S, Machado VM, MacIntyre MF, Magis-Rodriguez C, Mahdi AA, Majdan M, Malekzadeh R, Mangalam S, Mapoma CC, Marape M, Marcenes W, Margolis DJ, Margono C, Marks GB, Martin RV, Marzan MB, Mashal MT, Masiye F, Mason-Jones AJ, Matsushita K, Matzopoulos R, Mayosi BM, Mazorodze TT, McKay AC, McKee M, McLain A, Meaney PA, Medina C, Mehndiratta MM, Mejia-Rodriguez F, Mekonnen W, Melaku YA, Meltzer M, Memish ZA, Mendoza W, Mensah GA, Meretoja A, Mhimbira FA, Micha R, Miller TR, Mills EJ, Misganaw A, Mishra S, Mohamed IN, Mohammad KA, Mokdad AH, Mola GL, Monasta L, Montañez HJ, Montico M, Moore AR, Morawska L, Mori R, Moschandreas J, Moturi WN, Mozaffarian D, Mueller UO, Mukaigawara M, Mullany EC, Murthy KS, Naghavi M, Nahas Z, Naheed A, Naidoo KS, Naldi L, Nand D, Nangia V, Narayan KM, Nash D, Neal B, Nejjari C, Neupane SP, Newton CR, Ngalesoni FN, Ngirabega JD, Nguyen G, Nguyen NT, Nieuwenhuijsen MJ, Nisar MI, Nogueira JR, Nolla JM, Nolte S, Norheim OF, Norman RE, Norrving B, Nyakarahuka L, Oh I, Ohkubo T, Olusanya BO, Omer SB, Opio JN, Orozco R, Pagcatipunan RS, Pain AW, Pandian JD, Panelo CI, Papachristou C, Park E, Parry CD, Paternina CA, Patten SB, Paul VK, Pavlin BI, Pearce N, Pedraza LS (2015). Global, regional, and national comparative risk assessment of 79 behavioural, environmental and occupational, and metabolic risks or clusters of risks in 188 countries, 1990–2013: a systematic analysis for the Global Burden of Disease Study 2013. Lancet.

[3] Myers J, McAuley P, Lavie CJ, Despres J, Arena R, Kokkinos P (2015). Physical activity and cardiorespiratory fitness as major markers of cardiovascular risk: Their independent and interwoven importance to health status. Prog Cardiovasc Dis.

[4] Lee I, Shiroma EJ, Lobelo F, Puska P, Blair SN, Katzmarzyk PT, Lancet Physical Activity Series Working Group (2012). Effect of physical inactivity on major non-communicable diseases worldwide: An analysis of burden of disease and life expectancy. Lancet.

[5] Hallal PC, Andersen LB, Bull FC, Guthold R, Haskell W, Ekelund U, Lancet Physical Activity Series Working Group (2012). Global physical activity levels: Surveillance progress, pitfalls, and prospects. Lancet.

[6] Kohl HW, Craig CL, Lambert EV, Inoue S, Alkandari JR, Leetongin G, Kahlmeier S, Lancet Physical Activity Series Working Group (2012). The pandemic of physical inactivity: Global action for public health. Lancet.

[7] Guthold R, Stevens GA, Riley LM, Bull FC (2018). Worldwide trends in insufficient physical activity from 2001 to 2016: A pooled analysis of 358 population-based surveys with 1·9 million participants. Lancet Glob Health.

[8] Althoff T, Sosič R, Hicks JL, King AC, Delp SL, Leskovec J (2017). Large-scale physical activity data reveal worldwide activity inequality. Nature.

[9] Dennison BA, Erb TA, Jenkins PL (2002). Television viewing and television in bedroom associated with overweight risk among low-income preschool children. Pediatrics.

[10] Vale S, Santos R, Silva P, Soares-Miranda L, Mota J (2011). Relationship of objective measurement of physical activity during school hours and BMI in preschool children. Int J Pediatr Obes.

[11] Afshin A, Forouzanfar MH, Reitsma MB, Sur P, Estep K, Lee A, Marczak L, Mokdad AH, Moradi-Lakeh M, Naghavi M, Salama JS, Vos T, Abate KH, Abbafati C, Ahmed MB, Al-Aly Z, Alkerwi A, Al-Raddadi R, Amare AT, Amberbir A, Amegah AK, Amini E, Amrock SM, Anjana RM, Ärnlöv J, Asayesh H, Banerjee A, Barac A, Baye E, Bennett DA, Beyene AS, Biadgilign S, Biryukov S, Bjertness E, Boneya DJ, Campos-Nonato I, Carrero JJ, Cecilio P, Cercy K, Ciobanu LG, Cornaby L, Damtew SA, Dandona L, Dandona R, Dharmaratne SD, Duncan BB, Eshrati B, Esteghamati A, Feigin VL, Fernandes JC, Fürst T, Gebrehiwot TT, Gold A, Gona PN, Goto A, Habtewold TD, Hadush KT, Hafezi-Nejad N, Hay SI, Horino M, Islami F, Kamal R, Kasaeian A, Katikireddi SV, Kengne AP, Kesavachandran CN, Khader YS, Khang Y, Khubchandani J, Kim D, Kim YJ, Kinfu Y, Kosen S, Ku T, Defo BK, Kumar GA, Larson HJ, Leinsalu M, Liang X, Lim SS, Liu P, Lopez AD, Lozano R, Majeed A, Malekzadeh R, Malta DC, Mazidi M, McAlinden C, McGarvey ST, Mengistu DT, Mensah GA, Mensink GB, Mezgebe HB, Mirrakhimov EM, Mueller UO, Noubiap JJ, Obermeyer CM, Ogbo FA, Owolabi MO, Patton GC, Pourmalek F, Qorbani M, Rafay A, Rai RK, Ranabhat CL, Reinig N, Safiri S, Salomon JA, Sanabria JR, Santos IS, Sartorius B, Sawhney M, Schmidhuber J, Schutte AE, Schmidt MI, Sepanlou SG, Shamsizadeh M, Sheikhbahaei S, Shin M, Shiri R, Shiue I, Roba HS, Silva DA, Silverberg JI, Singh JA, Stranges S, Swaminathan S, Tabarés-Seisdedos R, Tadese F, Tedla BA, Tegegne BS, Terkawi AS, Thakur JS, Tonelli M, Topor-Madry R, Tyrovolas S, Ukwaja KN, Uthman OA, Vaezghasemi M, Vasankari T, Vlassov VV, Vollset SE, Weiderpass E, Werdecker A, Wesana J, Westerman R, Yano Y, Yonemoto N, Yonga G, Zaidi Z, Zenebe ZM, Zipkin B, Murray CJ, GBD 2015 Obesity Collaborators (2017). Health effects of overweight and obesity in 195 countries over 25 years. N Engl J Med.

[12] Ding D, Nguyen B, Learnihan V, Bauman AE, Davey R, Jalaludin B, Gebel K (2018). Moving to an active lifestyle? A systematic review of the effects of residential relocation on walking, physical activity and travel behaviour. Br J Sports Med.

[13] Kobel S, Kettner S, Kesztyüs D, Erkelenz N, Drenowatz C, Steinacker JM (2015). Correlates of habitual physical activity and organized sports in German primary school children. Public Health.

[14] Cliff DP, Okely AD, Morgan PJ, Jones RA, Steele JR, Baur LA (2012). Proficiency deficiency: Mastery of fundamental movement skills and skill components in overweight and obese children. Obesity (Silver Spring).

[15] Wijndaele K, Westgate K, Stephens SK, Blair SN, Bull FC, Chastin SF, Dunstan DW, Ekelund U, Esliger DW, Freedson PS, Granat MH, Matthews CE, Owen N, Rowlands AV, Sherar LB, Tremblay MS, Troiano RP, Brage S, Healy GN (2015). Utilization and harmonization of adult accelerometry data: Review and expert consensus. Med Sci Sports Exerc.

[16] Welk GJ, Kim Y (2015). Context of physical activity in a representative sample of adults. Med Sci Sports Exerc.

[17] Tudor-Locke C, Ham SA (2008). Walking behaviors reported in the American Time Use Survey 2003-2005. J Phys Act Health.

[18] Tudor-Locke C, Rowe DA (2012). Using cadence to study free-living ambulatory behaviour. Sports Med.

[19] Barreira TV, Katzmarzyk PT, Johnson WD, Tudor-Locke C (2012). Cadence patterns and peak cadence in US children and adolescents: NHANES, 2005-2006. Med Sci Sports Exerc.

[20] Tudor-Locke C, Han H, Aguiar EJ, Barreira TV, Schuna JM, Kang M, Rowe DA (2018). How fast is fast enough? Walking cadence (steps/min) as a practical estimate of intensity in adults: A narrative review. Br J Sports Med.

[21] Ortega FB PROFITH.

[22] Cadenas-Sánchez C, Mora-González J, Migueles JH, Martín-Matillas M, Gómez-Vida J, Escolano-Margarit MV, Maldonado J, Enriquez GM, Pastor-Villaescusa B, de Teresa C, Navarrete S, Lozano RM, de Dios Beas-Jiménez J, Estévez-López F, Mena-Molina A, Heras MJ, Chillón P, Campoy C, Muñoz-Hernández V, Martínez-Ávila WD, Merchan ME, Perales JC, Gil A, Verdejo-García A, Aguilera CM, Ruiz JR, Labayen I, Catena A, Ortega FB (2016). An exercise-based randomized controlled trial on brain, cognition, physical health and mental health in overweight/obese children (ActiveBrains project): Rationale, design and methods. Contemp Clin Trials.

[23] Cole TJ, Lobstein T (2012). Extended international (IOTF) body mass index cut-offs for thinness, overweight and obesity. Pediatr Obes.

[24] Bervoets L, Massa G (2014). Defining morbid obesity in children based on BMI 40 at age 18 using the extended international (IOTF) cut-offs. Pediatr Obes.

[25] van Hees VT, Gorzelniak L, Dean León EC, Eder M, Pias M, Taherian S, Ekelund U, Renström F, Franks PW, Horsch A, Brage S (2013). Separating movement and gravity components in an acceleration signal and implications for the assessment of human daily physical activity. PLoS One.

[26] van Hees VT, Sabia S, Anderson KN, Denton SJ, Oliver J, Catt M, Abell JG, Kivimäki M, Trenell MI, Singh-Manoux A (2015). A novel, open access method to assess sleep duration using a wrist-worn accelerometer. PLoS One.

[27] Migueles JH, Rowlands AV, Huber F, Sabia S, van Hees VT (2019). GGIR: A Research Community–Driven Open Source R Package for Generating Physical Activity and Sleep Outcomes From Multi-Day Raw Accelerometer Data. J Meas Phys Behav.

[28] Evenson KR, Catellier DJ, Gill K, Ondrak KS, McMurray RG (2008). Calibration of two objective measures of physical activity for children. J Sports Sci.

[29] Fairclough SJ, Taylor S, Rowlands AV, Boddy LM, Noonan RJ (2019). Average acceleration and intensity gradient of primary school children and associations with indicators of health and well-being. J Sports Sci.

[30] Delisle Nyström C, Pomeroy J, Henriksson P, Forsum E, Ortega FB, Maddison R, Migueles JH, Löf M (2017). Evaluation of the wrist-worn ActiGraph wGT3x-BT for estimating activity energy expenditure in preschool children. Eur J Clin Nutr.

[31] Gomez-Marcos MA, Recio-Rodríguez JI, Patino-Alonso MC, Martinez-Vizcaino V, Martin-Borras C, de-la-Cal-Dela-Fuente A, Sauras-Llera I, Sanchez-Perez A, Agudo-Conde C, García-Ortiz L, EVIDENT Study Investigators (2014). Relationship between physical activity and plasma fibrinogen concentrations in adults without chronic diseases. PLoS One.

[32] 2018 Physical Activity Guidelines Advisory Committee (2018). 2018 Physical Activity Guidelines Advisory Committee Scientific Report.

[33] Tudor-Locke C, Camhi SM, Leonardi C, Johnson WD, Katzmarzyk PT, Earnest CP, Church TS (2011). Patterns of adult stepping cadence in the 2005-2006 NHANES. Prev Med.

[34] De Craemer M, De Decker E, Santos-Lozano A, Verloigne M, De Bourdeaudhuij I, Deforche B, Cardon G (2015). Validity of the Omron pedometer and the actigraph step count function in preschoolers. J Sci Med Sport.

[35] Tudor-Locke C, Barreira TV, Schuna JM (2015). Comparison of step outputs for waist and wrist accelerometer attachment sites. Med Sci Sports Exerc.

[36] World Health Organization (2010). Global Recommendations on Physical Activity for Health.

[37] The Comprehensive R Archive Network (CRAN).

[38] Tudor-Locke C, Schuna JM, Han H, Aguiar E, Larrivee S, Hsia D, Ducharme S, Barreira TV, Johnson W (2018). Cadence (steps/min) and intensity during ambulation in 6-20 year olds: The CADENCE-kids study. Int J Behav Nutr Phys Act.

[39] Migueles JH, Cadenas-Sanchez C, Ekelund U, Delisle Nyström C, Mora-Gonzalez J, Löf M, Labayen I, Ruiz JR, Ortega FB (2017). Accelerometer data collection and processing criteria to assess physical activity and other outcomes: A systematic review and practical considerations. Sports Med.

[40] Tudor-Locke C, Brashear MM, Katzmarzyk PT, Johnson WD (2012). Peak stepping cadence in free-living adults: 2005-2006 NHANES. J Phys Act Health.

[41] Migueles JH, Tudor-Locke C, Löf M, Esteban-Cornejo I, Molina-Garcia P, Mora-Gonzalez J, Rodriguez-Ayllon M, Garcia-Marmol E, Ekelund U, Ortega FB, Cadenas-Sanchez (2019). Comparability of published cut-points for the assessment of physical activity: Implications for data harmonization. Scand J Med Sci Sports.

[42] Barreira TV, Schuna JM, Mire EF, Broyles ST, Katzmarzyk PT, Johnson WD, Tudor-Locke C (2015). Normative steps/day and peak cadence values for United States children and adolescents: National Health and Nutrition Examination Survey 2005-2006. J Pediatr.

[43] Barreira TV, Katzmarzyk PT, Johnson WD, Tudor-Locke C (2013). Walking cadence and cardiovascular risk in children and adolescents: NHANES, 2005-2006. Am J Prev Med.

[44] Martínez-Gómez D, Ruiz JR, Gómez-Martínez S, Chillón P, Rey-López JP, Díaz LE, Castillo R, Veiga OL, Marcos A, AVENA Study Group (2011). Active commuting to school and cognitive performance in adolescents: The AVENA study. Arch Pediatr Adolesc Med.

[45] Mendoza JA, Liu Y (2014). Active commuting to elementary school and adiposity: An observational study. Child Obes.

