# Supplementary material for: Step-Based Metrics and Overall Physical Activity in Children With Overweight or Obesity: Cross-Sectional Study
Source: JMIR Mhealth Uhealth. 2020 Apr 28;8(4):e14841. doi: 10.2196/14841 (PMC7218606; doi:10.2196/14841)
Supplement: Multimedia Appendix 1 [file mhealth_v8i4e14841_app1.docx]

**Table A1.** Anthropometry, sedentary time, time-based physical activity metrics, and step-based metrics of overweight/obese children stratified by weight status (according to the World Obesity Federation standards).

|  |  | Overweight  (n=26, 42% girls) | Mild obese  (n=46, 37% girls) | Severe obese  (n=21, 52% girls) | Morbid obese  (n=12, 33% girls) |
| --- | --- | --- | --- | --- | --- |
|  | Age (years) | 10.3 ± 1.0 | 10.3 ± 1.1 | 9.8 ± 1.2 | 9.2 ± 0.9 |
| Anthropometry | |  |  |  |  |
|  | Weight (kg) | 46.9 ± 7.3 | 57.5 ± 10.1 | 62.1 ± 10.2 | 64.3 ± 9.6 |
|  | Height (cm) | 142.7 ± 8.7 | 146.8 ± 8.4 | 143.4 ± 7.5 | 140.7 ± 5.9 |
|  | BMI (z-Score) | 2.18 ± 0.26 | 2.84 ± 0.31 | 3.68 ± 0.62 | 4.68 ± 0.82 |
| Waking and wearing time | |  |  |  |  |
|  | Waking time (min/day) | 919.8 ± 31.2 | 916.4 ± 32.4 | 915.7 ± 30.4 | 938.1 ± 26.8 |
|  | Wear time during waking (min/day) | 905.9 ± 39.9 | 897.9 ± 35.3 | 903.0 ± 31.7 | 917.3 ± 30.0 |
| Sedentary time and physical activity intensities | |  |  |  |  |
|  | Sedentary time (min/day) | 579 ± 73.3 | 610 ± 63.8 | 588.9 ± 78.3 | 633.2 ± 54.3 |
|  | Light intensity (min/day) | 287.6 ± 49.0 | 262.8 ± 49.8 | 284.9 ± 58.8 | 261.3 ± 44.2 |
|  | Moderate intensity (min/day) | 37.3 ± 12.4 | 33.4 ± 11.3 | 31.6 ± 9.5 | 33 ± 14.5 |
|  | Vigorous intensity (min/day) | 13.7 ± 7.7 | 10.2 ± 5.6 | 9.3 ± 7.8 | 8.6 ± 4.6 |
|  | MVPA (min/day) | 51 ± 17.9 | 43.6 ± 15.9 | 40.9 ± 16 | 41.6 ± 18.2 |
| Step-based metrics | |  |  |  |  |
|  | Volume (steps/day) | 9775.5 ± 2559.8 | 8467.8 ± 1903.1 | 8201.3 ± 1975.6 | 7920.9 ± 2232.3 |
|  | Peak 60 min (steps/min) | 69 ± 14.8 | 63.6 ± 11.5 | 59.9 ± 11.7 | 58.9 ± 18.2 |
|  | Peak 30 min (steps/min) | 83.5 ± 15.8 | 78.2 ± 12.1 | 74.1 ± 13.1 | 72.1 ± 19.4 |
|  | Peak 1 min (steps/min) | 116.9 ± 13.7 | 112.1 ± 11.1 | 107.8 ± 12.1 | 103.7 ± 17.4 |
|  | *Time spent at different cadence bands* |  |  |  |  |
|  | 0 steps/min (min/day) | 323.9 ± 71.4 | 355.5 ± 79.1 | 335.9 ± 88.1 | 380 ± 58 |
|  | 1-19 steps/min (min/day) | 444.2 ± 47.4 | 430.6 ± 69.6 | 453.6 ± 71 | 434.1 ± 56.1 |
|  | 20-39 steps/min (min/day) | 78.2 ± 24.9 | 69.2 ± 16 | 72.1 ± 11.5 | 68.2 ± 17 |
|  | 40-59 steps/min (min/day) | 31.7 ± 12.8 | 27.1 ± 7.8 | 25.5 ± 4.7 | 23.5 ± 8.3 |
|  | 60-79 steps/min (min/day) | 19.4 ± 9.3 | 15.8 ± 7.2 | 13.5 ± 5.2 | 13.1 ± 8.7 |
|  | 80-99 steps/min (min/day) | 11.8 ± 6.8 | 10.6 ± 5.5 | 9.1 ± 6.1 | 7.5 ± 7.2 |
|  | 100-119 steps/min (min/day) | 7.7 ± 5.3 | 6.7 ± 6.5 | 5.0 ± 5.0 | 6.7 ± 7.3 |
|  | > 120 steps/min (min/day) | 2.8 ± 3.6 | 1.2 ± 1.8 | 1 ± 1.4 | 1.5 ± 3.5 |

Data are presented as mean ± standard deviation. BMI: Body mass index; MVPA: Moderate-to-vigorous physical activity.
